# Special Issue “Plant Virus Epidemiology and Control”

**DOI:** 10.3390/v12030309

**Published:** 2020-03-12

**Authors:** Ioannis E. Tzanetakis, Robert Martin, Igor Koloniuk

**Affiliations:** 1 Department of Plant Pathology, Division of Agriculture, University of Arkansas System, Fayetteville, AR 72701, USA; 2 USDA-ARS, Horticultural Crops Research Unit, Corvallis, OR 97330, USA; 3 Department of Plant Virology, Institute of Plant Molecular Biology, Biology Centre of the Czech Academy of Sciences, v.v.i., 3301 České Budějovice, Czech Republic

We recently completed the Special Issue on ‘Plant Virus Epidemiology and Control’. As editors, we decided not to offer vouchers to scientists that submit to this issue. This action had an effect on the number of papers received and accepted. We received a total of 19 papers and we accepted four. 

The study of Kinoti et al. [1] provides a much-needed update on the status of Prunus viruses in Australia. The study was based on 100 trees from Prunus growing areas which were tested for 34 viruses and three viroids. Nine viruses and two viroids were detected, making this study the basis for decision-making on the movement of plant material across boarders.

The study of Tahzima et al. [2] focuses on two major Prunus viruses; Little cherry virus 1 and 2. In addition to studying the genetic diversity of the two viruses in Belgium, the authors discovered three other viruses that were never before reported in the country. The diversity of the two Little cherry viruses was significant, yet, there were no correlations between genotype or geography and the observed virus diversity.

The study of Dall et al. [3] deals with a major problem for the seed industry, viroid contamination. Using statistically based estimates of detection, the authors determined the sampling size needed to meaningfully detect viroids. The results of this study could lower testing costs without losing the ability to detect viroids in seed lots.

The fourth paper, the study of Alazem et al. [4], explores a different theme from the above-mentioned studies as it dissects the molecular interactions between an avirulent isolate of soybean mosaic virus and a susceptible soybean cultivar. Based on this study, it appears that in the absence of a strain-specific resistance gene which alters the effect of Abscisic acid on callose accumulation and the RNAi pathway, some soybean mosaic strains can reverse the effect of the hormone and are able to replicate and move within the plant.

Overall, the three editors are very satisfied with the quality of the published papers and we are looking forward to additional high-impact plant virus epidemiology and control papers being submitted to *Viruses*.
